# The acquisition order of leukemic drug resistance mutations is directed by the selective fitness associated with each resistance mechanism

**DOI:** 10.1038/s41598-023-40279-2

**Published:** 2023-08-11

**Authors:** Benjamin C. S. Leow, Chung H. Kok, David T. Yeung, Timothy P. Hughes, Deborah L. White, Laura N. Eadie

**Affiliations:** 1 Blood Cancer Program, Precision Cancer Medicine Theme, South Australian Health & Medical Research Institute, Adelaide, SA 5000 Australia; 2 Faculty of Health and Medical Sciences, University of Adelaide, Adelaide, SA 5000 Australia; 3 Australasian Leukaemia & Lymphoma Group, Richmond, VIC 3121 Australia; 4 Royal Adelaide Hospital, Adelaide, SA 5000 Australia; 5 Australian & New Zealand Children’s Haematology/Oncology Group, Clayton, VIC 3168 Australia; 6 Australian Genomics Health Alliance, Parkville, VIC 3052 Australia

**Keywords:** Cancer genetics, Cancer models, Evolutionary genetics, Cancer genomics, RNA sequencing, Chronic myeloid leukaemia, Cancer, Evolution

## Abstract

In Chronic Myeloid Leukemia, the transition from drug sensitive to drug resistant disease is poorly understood. Here, we used exploratory sequencing of gene transcripts to determine the mechanisms of drug resistance in a dasatinib resistant cell line model. Importantly, cell samples were collected sequentially during drug exposure and dose escalation, revealing several resistance mechanisms which fluctuated over time. BCR::ABL1 overexpression, BCR::ABL1 kinase domain mutation, and overexpression of the small molecule transporter ABCG2, were identified as dasatinib resistance mechanisms. The acquisition of mutations followed an order corresponding with the increase in selective fitness associated with each resistance mechanism. Additionally, it was demonstrated that ABCG2 overexpression confers partial ponatinib resistance. The results of this study have broad applicability and help direct effective therapeutic drug usage and dosing regimens and may be useful for clinicians to select the most efficacious therapy at the most beneficial time.

## Introduction

The introduction of tyrosine kinase inhibitor (TKI) treatment for diseases driven by aberrant BCR::ABL1 kinase activity has revolutionized patient survival. In Chronic Myeloid Leukemia (CML), the use of the 1st generation TKI imatinib to target the cancer driver, BCR::ABL1, results in 10 year survival rates of over 83%, approaching that of the healthy age-matched population^1^. Suboptimal response however, occurs in 10–15% of imatinib treated patients, and in < 10% of patients treated with 2nd generation TKIs nilotinib, dasatinib and bosutinib^2^. The generation of drug resistant tumor cell lines by in vitro exposure to chemotherapy provides an experimental system recapitulating therapy resistance. This methodology has often been used to study the resistance of CML to both traditional chemotherapeutics^3,4^, as well as BCR::ABL1 targeted TKI treatment^5–16^. Using gradual escalation of TKI in cell culture media, these models are able to recapitulate molecular mechanisms of drug resistance observed in the clinic, including alteration of the drug target BCR::ABL1, perturbed expression of cell membrane transporters, downregulation of apoptosis, and activation of alternate cancer drivers. Alternatively, niche factors such as stromal support or cellular quiescence induced by the bone marrow microenvironment allow leukemic cells to evade apoptosis and promote relapse^17^. A malignancy will commonly respond to treatment with an initial reduction in tumor burden, only for a population of persisting tumor cells to later repopulate the disease. In CML, relapse often originates from a small number of inherently resistant cells within the tumor cell population, harboring a degree of TKI resistance, even prior to drug selection^18,19^. Prolonged therapy administration selects for these resistant characteristics, resulting in overt drug resistance.

The first imatinib resistance mechanism identified was overexpression of the BCR::ABL1 fusion protein^6^. Increased expression of BCR::ABL1 alters the balance between kinase inhibition due to competitive kinase inhibitors, and increased protein activity, restoring proliferative and anti-apoptotic signaling. The resultant disease has an inherent resistance to not just imatinib, but also 2nd and 3rd generation of BCR::ABL1 inhibitors, such as nilotinib, dasatinib and ponatinib^8,16,20^. Upregulation of BCR::ABL1 expression can occur at each regulatory stage, including DNA gene amplification^21,22^ or overexpression of the gene transcript and protein^23^. Drug efflux resistance mechanisms are characterized by the increased expression of ATP-binding cassette (ABC) transporters, such as ABCB1 (P-glycoprotein, MDR-1) or ABCG2 (BCRP), which are able to pump a variety of small molecules out of the cancer cell in an ATP dependent manner^11,24,25^. Conversely, the reduced functional activity of cell membrane solute carrier transporters, such organic cation transporter (OCT-1), can also be associated with a lack of TKI response^26,27^. Mutations within the ABL kinase domain are the most commonly identified resistance mechanism. They lower drug binding affinity to BCR::ABL1 and confer varying levels of resistance^28,29^. In particular, the p.T315I mutation confers complete resistance to imatinib, nilotinib, dasatinib and bosutinib, though 3rd generation ponatinib, which was developed to target the p.T315I mutation, has clinical activity^10^.

A number of reports have previously modeled resistance mechanisms by encouraging outgrowth of resistant cells through gradual escalations in drug exposure. However, few of these have examined clonal evolution in intermediary lines with incremental increases of drug exposure. Additionally, studies often use only targeted assays for putative resistance mechanisms, and are likely to overlook uncommon or unexpected factors. Consequently, these studies may miss population dynamics, and omit the cellular adaptations that arise early in drug exposure, which facilitate subsequent, more overt drug resistance. Determining the factors contributing to low level drug resistance, and the evolutionary trajectory of the tumor cell population, may prevent resistance mutation development by informing possible concomitant drug combinations and treatment dosing regimens to deliver optimal drug efficacy.

We hypothesized that a cell lineage having a diverse array of resistance mechanisms is of relevance, and that during treatment, cells harboring the most advantageous resistance mechanisms (highest TKI resistance) would be selected for over time. We comprehensively examined retrospective samples from a dasatinib-resistant CML cell line, K562 DasR, previously established by our laboratory^20^. The TKI dasatinib was initially developed for use in imatinib resistance, but has subsequently entered clinical use in the frontline setting^30^. The BCR::ABL1-expressing K562 line was cultured in gradually increasing concentrations of dasatinib, with samples examined at each stage of resistance generation to interrogate the dynamics of TKI resistance development. RNAseq transcriptome profiling was used to identify changes in global gene expression over the course of resistance generation. We observed the BCR::ABL1 overexpression and the p.T315I mutation previously reported in the resistant cell line^20^. In addition, the overexpression of drug transporter ABCG2 was identified in a subset of K562 DasR cells, and its contribution to dasatinib and ponatinib resistance was determined. Our study revealed a distinct order in the evolution of drug resistant mutations, recapitulating the evolutionary dynamics observed in CML patients. Using these observations, we developed the ‘resistance mechanism associated burden’ hypothesis. Our hypothesis accurately interprets the rise and fall of tumor cell populations expressing varied TKI resistance mechanisms observed here, and may have broad applicability across several fields of therapy resistance research.

## Results

### Differential expression analysis reveals potential mediators of dasatinib resistance

To identify the cellular mechanism(s) contributing to TKI resistance, mRNA transcriptome sequencing was performed on K562 10 nM, 15 nM, 25 nM, 50 nM and 200 nM DasR intermediates, as well as drug naive and DMSO vehicle control lines. Results from unsupervised hierarchical clustering showed a high degree of similarity in the K562 10, 15 and 25 nM DasR (early stage) intermediates, which clustered separately from the 50 and 200 nM DasR (late stage) intermediates and control lines (Supplementary Fig. 1). Differential expression analysis was thus performed between the early stage intermediates and K562 control lines, and between early and late stage intermediates, visualized by volcano plot (Supplementary Fig. 2). Between the early stage intermediates and control lines, there were 435 genes significantly differentially expressed (FDR < 0.005). Of these, a number of genes with increased expression were putative candidates for TKI resistance. *SPP1* (encoding osteopontin)^31,32^, *MAPK4*^33^, *SMO* (encoding smoothened)^34,35^ and *FZD3* (encoding frizzled protein 3)^36^ have known function as cancer progression promoters. Furthermore, there was down-regulation of tumor suppressor gene *RBM47*^37^, and the apoptotic regulator, *G0S2*^38,39^.

Differential gene expression results were further analyzed by geneset enrichment analysis (GSEA). Within the MSigDB Hallmark genesets (Supplementary Table 2a), upregulation of MYC signaling was identified in early stage intermediates, which was downregulated in late stage intermediates (Supplementary Fig. 3). This is likely downstream of increased BCR::ABL1 expression and kinase signaling. Within the C5 gene ontology genesets (Supplementary Table 2b), upregulation of ribosome associated genes was identified in early stage intermediates, which were again downregulated in late stage intermediates. This is possibly due to the increased anabolic requirements of early intermediates overexpressing BCR::ABL1 or ABCG2 as a resistance mechanism, compared with the later T315I mutated intermediates requiring fewer cellular resources to maintain drug resistance.

**Figure 1:**
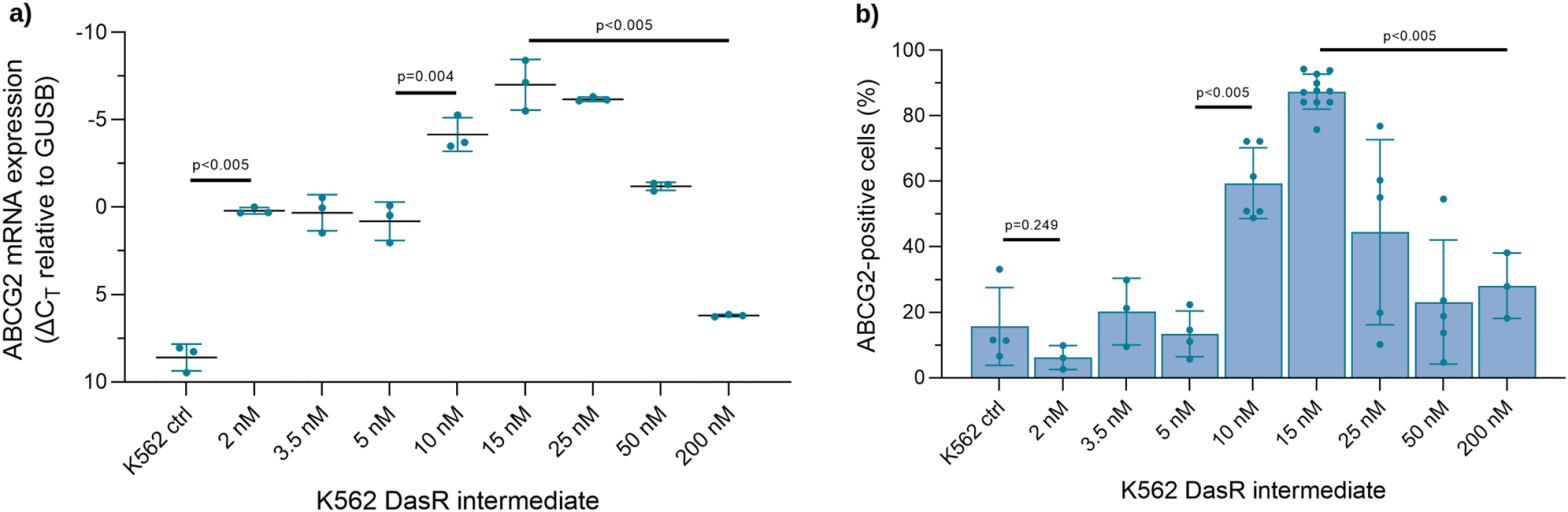
Significantly increased *ABCG2* expression was observed following exposure to dasatinib. K562 DasR intermediate cell lines were evaluated for expression of *ABCG2* (**a**) gene transcript (**b**) surface protein. Gene expression was determined by RT-PCR. Data were normalized to expression of housekeeping gene *GUSB* and expressed as the difference in mean Ct value (ΔCt) + /– SD. All data are representative of a minimum of 3 independent biological replicates. Significance was determined by Student’s *t*-test with Welch’s correction.

Importantly, the *ABCG2* small molecule membrane transporter gene was identified as highly overexpressed in K562 DasR early dose escalation intermediates, compared with controls (log fold change = 10.388, FDR = 1.363 × 10^–11^, Supplementary Fig. 2). Gene expression levels of *ABCG2* were quantified by confirmatory RT-PCR, showing an increase in *ABCG2* mRNA expression in all K562 DasR intermediates versus K562 DMSO control line (Fig. 1a). *ABCG2* gene expression in the 2 nM DasR intermediate was significantly higher than control (ΔCt = 0.209 vs 8.587, p < 0.005). Expression again increased between the 5 and 10 nM intermediates (ΔCt = 0.807 vs − 4.157, p = 0.004), before significantly decreasing between the 15 and 200 nM DasR lines (ΔCt = − 7.014 vs 6.194, p < 0.005).

To confirm that *ABCG2* mRNA was also expressed as a functional protein, surface expression of ABCG2 was determined by flow cytometry (Fig. 1b). ABCG2 flow cytometry differed slightly from RT-PCR results; with no significant increase in the K562 2, 3.5, and 5 nM DasR intermediates, compared with DMSO control. The first intermediate to demonstrate significantly higher ABCG2 protein expression was K562 10 nM DasR (13.4% vs 59.4%, p < 0.005), peaking with the 15 nM DasR intermediate (87.4%), before falling in the final 200 nM DasR line (28.1%, p < 0.005). The increased expression of ABCG2 in the 15 nM DasR intermediate remained stable over the course of ~ 7 months of continuous culture in 15 nM dasatinib with flow cytometric data indicating a single population of cells with high ABCG2 expression. However, a bi-modal population of low and high ABCG2 expressing cells was clearly observed in 3.5 nM and 10 nM DasR intermediates. These data indicate that cells with increased ABCG2 expression in the 3.5 nM and 10 nM DasR intermediates had a growth advantage and were selected for during dasatinib dose escalation. Conversely, when cells were cultured in higher concentrations of dasatinib > 15 nM, there was a deselection of cells with increased ABCG2 expression (Supplementary Fig. 4).

**Figure 2:**
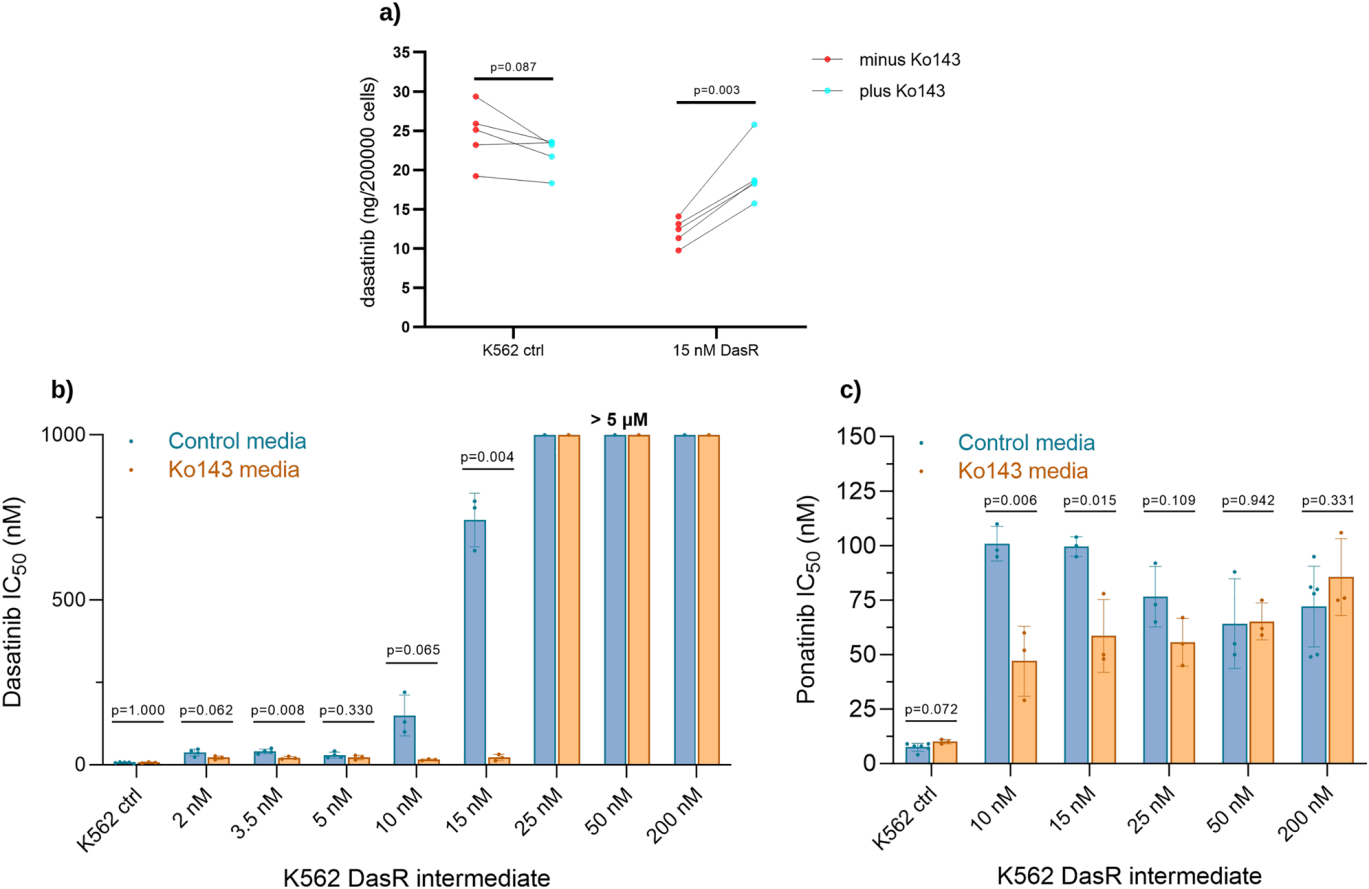
Dasatinib resistant cells are sensitized to dasatinib/ponatinib with co-administration of ABCG2 inhibitor, Ko143. (**a**) Dasatinib uptake and retention was determined in selected K562 DasR lines, in the presence and absence of ABCG2 inhibitor, Ko143. Significance was determined by paired sample *t* test. (**b**) Dasatinib IC_50_ and (**c**) ponatinib IC_50_ were determined in the absence (blue bars) and presence (orange bars) of Ko143. IC_50_ was determined by measuring phosphorylation of BCR::ABL1 kinase target, CrkL, and expressed as mean + /– SD. All data are representative of a minimum of 3 independent biological replicates. Significance was determined by paired sample *t*-test.

The correlation between ABCG2 expression and dasatinib cellular efflux was confirmed using the dasatinib IUR assay in the presence versus absence of the ABCG2 inhibitor, Ko143 (Fig. 2a). We compared K562 control versus 15 nM DasR cells as a representative, as this intermediate demonstrated greatest ABCG2 expression (Fig. 1). Dasatinib uptake in K562 control without Ko143 was significantly higher than in K562 15 nM DasR cells (24.56 vs 12.15 ng/200,000 cells, p = 0.0007). The addition of Ko143 to control cells had no significant effect on dasatinib uptake (22.07 vs 24.5 ng/200,000 cells, p = 0.087). However, in K562 15 nM DasR cells, treatment with Ko143 significantly increased dasatinib retention (12.15 vs 19.39 ng/200,000 cells, p = 0.003), confirming ABCG2-mediated dasatinib efflux as a resistance mechanism.

### Inhibition of ABCG2 activity leads to reversal of dasatinib resistance, as well as ponatinib sensitization

To quantitate the level of TKI resistance at each stage of dasatinib dose escalation, the p-CrkL IC_50_ measure was used. As expected, results indicated a gradual increase in dasatinib IC_50_ coinciding with exposure to dasatinib and dose escalation (Fig. 2b, blue bars). Between the K562 DMSO control line and the 2 nM DasR intermediate, in the absence of Ko143, dasatinib IC_50_ increased from 7 to 37.5 nM (p = 0.047). Dasatinib IC_50_ was relatively stable until the 10 nM DasR intermediate, where it increased to 150 nM (p = 0.024), followed by a further increase to 743 nM, indicating overt resistance (p = 0.001) in the 15 nM DasR intermediate. In the 25 nM DasR intermediate and beyond, dasatinib-mediated inactivation of BCR::ABL1 activity was not possible, even when cultured in 5000 nM dasatinib, which is ~ 25 × the maximum achievable blood plasma concentration^40^.

We confirmed that, in the presence of ABCG2 as the primary resistance mechanism, using Ko143 to block drug efflux could restore TKI sensitivity through increased intracellular drug retention (Fig. 2b). While the addition of Ko143 had no effect on dasatinib sensitivity in the K562 DMSO control line (7 vs 7 nM dasatinib, p = 1), the addition of Ko143 resulted in a reduction in dasatinib IC_50_ in K562 early stage DasR intermediates. This effect was most pronounced in the 15 nM DasR intermediate which demonstrated the highest level of ABCG2 expression (743 vs 22.3 nM dasatinib, p = 0.004). In the 25, 50 and 200 nM DasR lines, the addition of Ko143 had no effect, suggesting the evolution of an additional, ABCG2 independent resistance mechanism (later demonstrated to be the emergence of p.T315I, below).

In cases of dasatinib treatment failure, the 3rd generation TKI ponatinib is often prescribed as salvage therapy, in particular due to its activity inhibiting BCR::ABL1 mutant, p.T315I. We demonstrated that ABCG2 also had relevance for ponatinib resistance in intermediate cells with highest expression, as demonstrated using IC_50_ assays. Ponatinib IC_50_ assay was performed in a subset of K562 DasR intermediates, selected based on ABCG2 expression (Fig. 2c). All K562 DasR lines had a ponatinib IC_50_ higher than K562 control. It was demonstrated that in the 10 and 15 nM DasR cells, expressing the highest ABCG2 levels, the addition of Ko143 had a ponatinib sensitizing effect; addition of Ko143 in the K562 15 nM DasR intermediate reduced ponatinib IC_50_ from 99.7 nM to 58.7 nM (p = 0.015), and from 101 to 47 nM in the K562 10 nM DasR intermediate (p = 0.006). In K562 later stage DasR intermediates with lower ABCG2 expression, Ko143 elicited no significant change to ponatinib IC_50_, nor in the DMSO control line.

### Resistance to dasatinib is conferred by both BCR::ABL1 overexpression and p.T315I mutation during K562 DasR dose escalation

**Figure 3:**
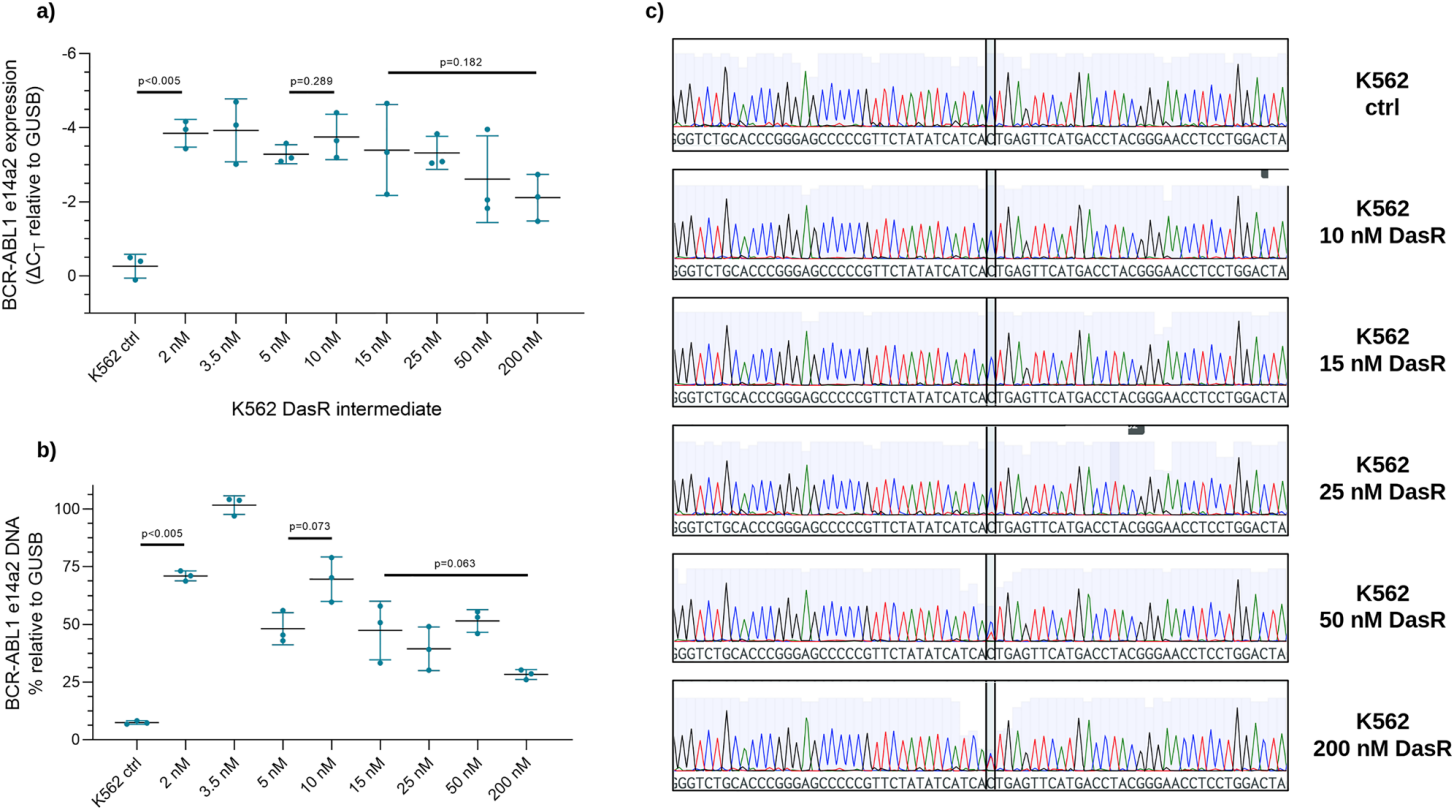
*BCR::ABL1* transcript expression and copy number rise and fall over dasatinib exposure. (**a**) *BCR::ABL1* gene transcription and (**b**) DNA copy number in K562 DasR intermediates was determined by qPCR. Transcription data is presented as ΔCt relative to *GUSB*, and DNA quantity is presented as a percentage of *GUSB* gene amplification. Both data are reported as mean –/ + SD. Significance was determined by Student’s *t*-test with Welch’s correction. (**c**) Sanger sequencing of the *BCR::ABL1* kinase domain was performed in K562 DasR intermediates. Highlighted region indicates the nucleotide location p.T315, with the p.T315I mutation first detectable in the K562 25 nM Das intermediate, and expanding with increasing TKI exposure.

Preliminary targeted characterization of putative resistance mechanisms identified two factors contributing to dasatinib resistance: the increased expression of *BCR::ABL1* mRNA in early dasatinib dose escalation intermediates, and mutation of the p.T315I residue, first identified in the 25 nM DasR intermediate^20^; these findings were validated here. *BCR::ABL1* mRNA transcript levels were determined by RT-PCR (Fig. 3a). Results indicated a significant increase in *BCR::ABL1* expression compared with expression in control cells occurring early in dasatinib dose escalation: control vs 2 nM DasR intermediate (ΔCt = − 0.267 vs − 3.587, p < 0.005). *BCR::ABL1* expression then stabilized, before trending a reduction, reaching a minimum in the final 200 nM DasR resistant line (15 vs 200 nM DasR intermediate, ΔCt = − 3.403 vs − 2.120; p = 0.182). It was noted that *BCR::ABL1* expression results here differed marginally from those obtained previously; this is likely due to the different primer targets and advances in technology.

To determine whether *BCR::ABL1* mRNA fluctuations were driven by changes in DNA copy number, qPCR for genomic *BCR::ABL1* sequences was performed in K562 DasR dose escalation intermediates (Fig. 3b). A significant increase in genomic *BCR::ABL1* was observed in the 2 nM DasR compared with K562 control (71% vs 8%, p < 0.005). *BCR::ABL1* quantity further increased in the 3.5 nM intermediate (102%, p < 0.005). Analogous to *BCR::ABL1* gene expression, copy number peaked in the 3.5 nM DasR intermediate (102%), before decreasing, reaching a minimum in the K562 200 nM DasR line (28%). DNA amplification was also confirmed by FISH in the K562 200 nM DasR line, as compared with DMSO control (data not shown).

The contribution of *BCR::ABL1* mutations to resistance was determined by Sanger sequencing, performed at each stage of TKI resistance generation (Fig. 3c). Results demonstrated the presence of the p.T315I mutation in the 25, 50, and 200 nM DasR intermediates, representing approximately 2%, 30%, and 50% of *BCR::ABL1* transcripts respectively. In K562 DasR intermediates prior to K562 25 nM DasR, no mutations were detected. The emergence of p.T315I, which conferred high level resistance to dasatinib, coincided with a relative decrease in ABCG2 expression, a mechanism of resistance no longer contributory.

## Discussion

**Figure 4:**
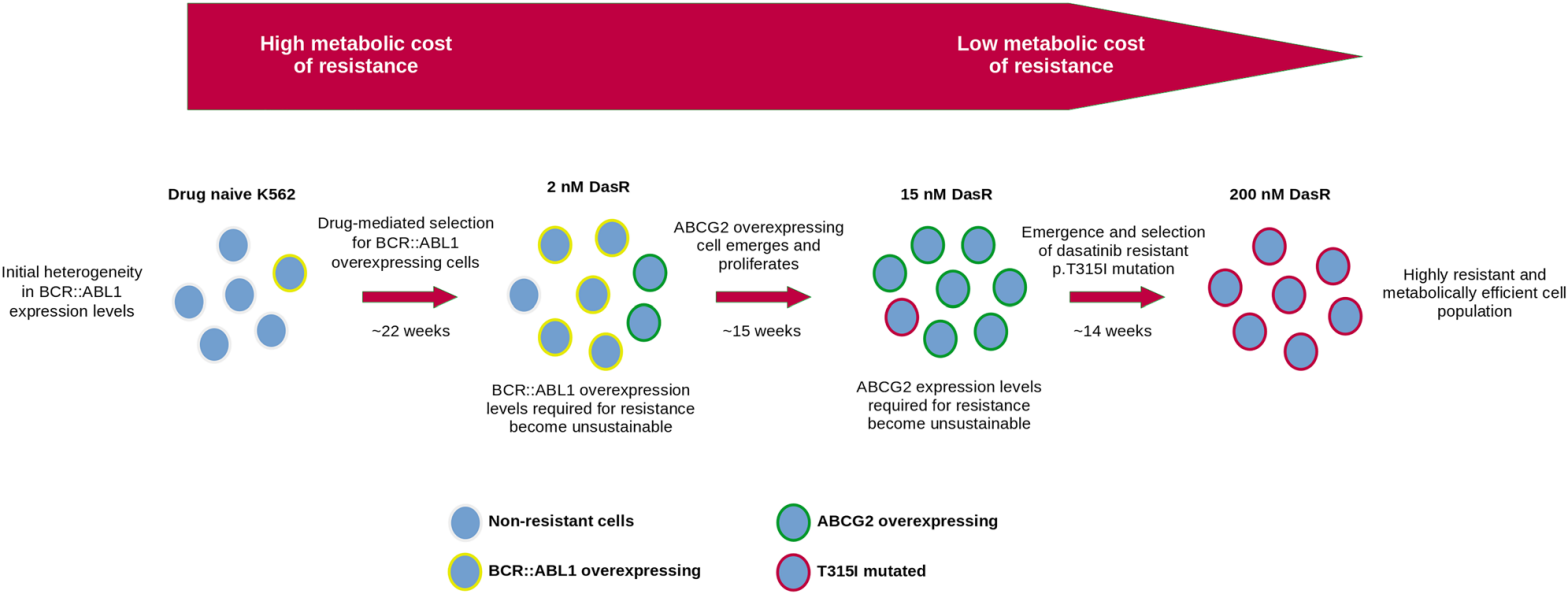
Schematic representation of the ‘resistance mechanism associated burden’ hypothesis. Summary of the principal TKI resistance mechanisms at each stage of dasatinib resistance generation. The transition from resistance mechanisms of high to low metabolic cost formed the basis of the hypothesis proposed in this study.

Our study identified and validated three separate TKI resistance mechanisms, and tracked them over time to observe the selection and de-selection of an evolving cell population. While more complex to interrogate, our study more completely describes the dynamics of drug resistant disease. Often, the prolonged treatment of a cancer cell population with targeted therapies results in a loss of treatment efficacy, and ascertaining and abrogating the mechanism(s) of treatment resistance may re-sensitize the tumor^41^. Here, we observed that several populations of tumor cells, each harboring distinct resistance mechanisms, can be simultaneously present, fluctuating temporally and evidencing a Darwinian evolutionary trajectory (Fig. 4). Several models exist for the dynamics of cell competition, whether by a linear chain of accrued mutations with a single ancestor, a branching/neutral evolution of coexisting genotypes and phenotypes, or punctuated equilibrium whereby a selective sweep occurs preferencing a particularly fit cell subpopulation^42^. Indeed, features of all these models were observed in our study. In the context of drug exposure, the selective fitness of a cancer cell is largely determined by its ability to maintain proliferative and anti-apoptotic signaling. The mechanism(s) by which it achieves this, i.e. driver gene overexpression, or expression of ATP-consuming drug transporters, appear to have a biological fitness cost associated with them. The high level of *BCR::ABL1* expression observed in early K562 DasR intermediates requires each cell to synthesize ~ tenfold more BCR::ABL1 protein than the drug naive control, a costly energetic burden given the ATP and GTP expense associated with protein assembly and tRNA synthesis^43,44^. There is also the possibility that perturbations caused by these resistance mechanisms is disruptive to regular cell function and replication, resulting in decreased selective fitness. Supporting this theory, a reduction in selective fitness has been associated with cases of bacterial antibiotic resistance^45^, as well as weed herbicide and insect pesticide resistance^46^. In our study, the gene expression and amplification of *BCR::ABL1* was initially increased in early stage resistance intermediates, however, following the emergence of other resistance mechanisms, *BCR::ABL1* levels decreased in later resistance intermediates. The observed reduction of expression could be explained by a selective fitness cost associated with increased *BCR::ABL1* as a resistance mechanism, and selection against this cell population via outcompetition by fitter cells. Assessment of the population fluctuations in the TKI resistant cells underlies the hypothesis that the specific cellular cost associated with each resistance mechanism, balanced against the level of TKI resistance provided, is responsible for the resultant selective fitness and observed population dynamics.

Our study demonstrated that ABCG2-mediated drug efflux was a significant driver of dasatinib resistance, in agreement with studies from our laboratory and others^47–49^. Nevertheless, in our model TKI resistance mediated by ABCG2 transporter expression was selected for in favor of BCR::ABL1 overexpression. There are several potential factors contributing to this. Detection of *ABCG2* mRNA increases in the K562 2, 3.5 and 5 nM DasR intermediates indicate the likely presence of a small subpopulation of cells expressing ABCG2, appearing early in dasatinib treatment. However, ABCG2 surface protein expression was only detected following further dose increases to 10 and 15 nM dasatinib, suggesting that the selective advantage of ABCG2 expression occurs mostly at these higher dasatinib concentrations. The high-level expression of the ABCG2 transporter likely incurred a significant fitness cost. Previously, attempts have been made to quantify the cellular energy expenditure associated with ABC transporter expression. MCF-7 breast cancer cells expressing ABCB1 as a resistance mechanism have been observed to exhibit higher glycolytic metabolism than their parental counterparts, reducing their selective fitness^50^, and it is probable that the ABCG2 expression observed in K562 DasR intermediates imposed a similar fitness cost. However, this cost appears lower than the metabolic cost of overexpressing BCR::ABL1 as a resistance mechanism; once translated, each ABCG2 transporter is able to transport many dasatinib molecules out of the cancer cell cytosol, whereas for resistance mediated by BCR::ABL1 overexpression alone, an entire BCR::ABL1 protein must be translated to counteract each TKI molecule. Additionally, the half-life of ABCG2 is somewhat longer than that of *BCR::ABL1* (~ 54 h vs ~ 40 h)^51,52^. It is thus likely that drug efflux is generally a more energy efficient resistance mechanism than driver gene overexpression. If both of these resistance mechanisms are capable of overcoming a given drug concentration, it is likely that the more energy efficient mechanism will be selected for.

A similar comparison can be made between ABCG2-mediated drug transport and BCR::ABL1 kinase domain mutation. The p.T315I mutation results in complete dasatinib resistance, whereas there appears to be an upper limit to the TKI concentration that can be overcome by increased ABC transporter expression alone. While the degree of resistance to kinase inhibition varies by mutation and according to the TKI used^53^, there is no theoretical energy cost associated with mutation, which may help explain our finding that cells harboring p.T315I are selected for, eventually reaching clonal dominance. This is similar to findings from previous TKI resistance studies in our laboratory, in which overexpression of the ABCB1 transporter preceded emergence of ABL1 p.V299L and p.F359C mutations in dasatinib and imatinib resistance models respectively^11^ and both ABCB1 and ABCG2 transporter overexpression preceded p.F497L emergence in an asciminib resistance model^14^. Adding to the complexity of this observation, some mutations in BCR::ABL1 and other kinases result in altered proliferative signaling. Multiple studies have interrogated the function of common BCR::ABL1 kinase domain mutants, finding that mutation alters kinase activity, transformation potential and kinase substrate specificity, and can act as both gain-of-function or loss-of-function. Studies of p.T315I have inconsistently determined this mutation to be either kinase-activating^54,55^ or inactivating^56,57^. Our own preliminary experiments have determined that following emergence of p.T315I under dasatinib selection, p.T315I levels decrease following prolonged dasatinib withdrawal, suggesting that cells harboring p.T315I have a selective disadvantage in the absence of TKI exposure (unpublished data). In lung cancer studies, TKI resistant mutants of EGFR kinase exhibit lower proliferation than wildtype EGFR^58^. However, regardless of the effect of the p.T315I mutation on kinase activity, presence of the mutation retains BCR::ABL1 activity while conferring high-level TKI resistance.

Findings of the study presented here suggest that ABCG2 can also mediate ponatinib efflux from the leukemic cell. Three additional studies have explored transport of ponatinib by ABCG2. Two of these studies found ponatinib to be a transported substrate of ABCG2^59,60^ the third, conducted by our own laboratory, concluded ponatinib transport is not mediated by ABCG2^61^. Sen et al. found ponatinib induced ATPase activity in ABCG2 transfected leukemic cells, which also exhibited lowered ponatinib sensitivity/higher IC_50_, while also acting as an ABCG2 inhibitor^59^. Similarly, Kort et al. determined ABCG2 contributed to lower accumulation of ponatinib into the mouse brain, as well as transporting ABCG2 in vitro^60^. Conversely, Lu et al. concluded that ABCG2 overexpression does not increase ponatinib IC_50_, nor does ABCG2 inhibition decrease ponatinib IC_50_^61^. Excessive activity of the ABC transporters can drive TKI resistance, although there is considerable debate as to whether particular molecules act as substrate or inhibitor, and the clinical relevance of TKI concentrations used in experimental systems^62^. The modest level of ponatinib transport conferred by ABCG2 measured by the former studies may mean detectable ponatinib transport may only occur at high ABCG2 expression levels; discrepancies in results may be due to these differences between model systems. Alternatively, polymorphisms in the ABCG2 sequence have been observed to alter transporter function^63^. Validation of experimental systems may help clarify observations and avoid inconsistency in conclusions.

In our model, some additional questions remain as to the role of ABCG2, as well as its transcriptional control. Previous studies have demonstrated that ABCG2 expression is associated with a stem cell phenotype, and may be a determinant of this phenotype^64^. While we have identified genes in our differential expression analysis known to be involved in stemness, it is not known whether the increased ABCG2 expression observed here was upstream, downstream, or unrelated with stem cell maintenance. We did not perform ABCG2 DNA quantity analysis, which may have indicated gene amplification as a pathway to transcript overexpression. Alternatively, it has been demonstrated recently that expression of ABCG2 is induced in the presence of xenobiotics^65^, another pathway which may have been active in our resistance model.

The exploratory transcriptome sequencing performed on K562 DasR dose escalation intermediates identified several genes as potentially contributing to TKI resistance. While the sample size was small and the results require validation, it was noted that a number of the genes have previously been associated with cancer therapeutic resistance. Early K562 DasR intermediates overexpressed genes encoding activating proteins involved in the MAPK pathway governing cellular proliferation and differentiation^66^, as well as the gene encoding osteopontin, which has a known role in CML progression^31,32,67^. These transcriptomic aberrations are likely downstream of increased BCR::ABL1 function. The gene *FZD3* was also overexpressed, a cognate receptor for Wnt signaling with known roles in cancer^36,68^, as well as the gene encoding Smoothened protein, an important Hedgehog signaling pathway member which regulates cell fate and maintain drug resistance in CML stem cells^34,35^. The protein product of the overexpressed *PM1K* gene is also involved in hematopoietic cell maintenance and leukemogenesis^69^. It is possible that expression of these genes was upregulated, allowing cells with drug resistant stem cell-like properties to emerge. Furthermore, reduced expression of the *G0S2* switch gene was observed in early stage resistant cells compared with control. The protein product of *G0S2* canonically interacts with the apoptotic Bcl2 protein, which may have allowed a protective lowering of apoptosis^38,39,70^. G0S2 activity also results in a differentiation block and TKI resistance in CML cells^39^. To analyze gene expression data in a more systematic way, GSEA analysis was performed. While the findings of deregulated MYC signaling in dasatinib resistant cells was interesting, it was likely a reflection of BCR::ABL1 expression and activity in these cells. Intriguingly, transcriptional control of MYC lies both upstream^71^ and downstream^72^ of BCR::ABL1; it is unknown here which event first occurred. GSEA analysis also identified several genesets relating to ribosomal activity, again upregulated in early dasatinib intermediates and downregulated in later intermediates. Assuming this is downstream of resistance mechanism gene expression, these data support our hypothesis regarding drug resistance mechanisms and their detrimental effect on cellular function and selective fitness.

It is also worth mentioning that in addition to BCR::ABL1, dasatinib is also an inhibitor of several other protein kinases, including KIT, PDGFRβ, HCK, LYN, wildtype ABL kinase, as well as other SRC family kinases^73^. It is highly likely that the inhibition of these kinases by dasatinib would have an effect on the gene expression of the dasatinib resistant cells, and compensatory upregulation of these pathways is also likely. Nevertheless, the differentially expressed genes identified here did not confer BCR::ABL1 independent proliferation, and inhibition of BCR::ABL1 activity resulted in cell death in all K562 DasR lines, indicating that BCR::ABL1 remained the dominant driver of malignancy. In late stage DasR intermediates, fewer resistance related genes demonstrated altered expression, suggesting the homogeneity of leukemic cells harboring the highly resistant p.T315I mutation.

One limitation of the study performed here, and an opportunity for further study, is consideration of the role of the cellular microenvironment in supporting TKI resistance. The bone marrow microenvironment is comprised of a vast array of cells, including endothelial cells, fibroblasts, and multipotent mesenchymal cells, with their chondro-, adipo-, and osteo-blastic progeny^74,75^. The environment changes in response to acute infection and inflammation, as well as age, and presence of malignancy and/or xenobiotics^76^. Bone marrow stromal support protects CML stem cells from TKI-induced cell death, enhancing maintenance and lowering apoptosis^68^. Previous studies have demonstrated dasatinib is able to remodel bone tissue through osteoclast inhibition^77^ and osteogenic differentiation^78^. It is likely that the selective pressure of long term dasatinib treatment also affects bone marrow niche architecture, and has marked effects on TKI treatment. How this specifically functions remains to be elucidated.

The most important outcomes of this study relate to patient care, and determining ways to effectively manage CML treatment and maximize TKI efficacy. While relatively simplistic, in vitro TKI dose escalation models effectively model many aspects of TKI resistance. CML cell lines, derived from patients and driven by BCR::ABL1 activity, accurately recapitulate in vivo leukemic cells, and the resistance mechanisms observed in patients. Nevertheless, some caveats of the dose escalation model remain. The majority of CML cell lines were derived from blast crisis CML patients, and exhibit a high level of mutability, likely higher than in most chronic phase CML cases. Optimal drug delivery minimizes drug resistance in CML, and the levels of TKI resistance observed in in vitro models is higher than that observed in patients. The low level starting drug exposure in our dose escalation model is perhaps more reminiscent of patient cases having inconsistent dosing, problematic patient specific pharmacokinetics, or niche factors affecting drug delivery to target cells. Nevertheless, the dynamics of resistance mechanism fluctuations observed in our experiments are comparable with that observed in patients. The association of a selective burden with the development of some resistance mechanisms has important therapeutic implications. It has been previously suggested that chemotherapeutic treatment regimens should have an evolution based strategy, such as temporarily halting chemotherapy treatment in drug resistant patients to allow resurgence of drug susceptible cell populations and subsequent treatment efficacy^79,80^, or alternating multiple chemotherapeutics to exploit population genetic homogeneity^81^. With regard to cell membrane transporter mediated resistance, a number of specific potential therapies have been suggested. It may be possible to preempt emergence of ABC transporter overexpression by utilizing combination therapy with transporter inhibitors^82^. The complex TKI:ABC transporter relationship where some TKIs function as both substrate and inhibitor may be exploited to simultaneously inhibit BCR::ABL1 kinase activity and drug efflux, with benefit maximized by combining lower doses of multiple TKIs^83^ or other chemotherapeutics^84^. However, our data also demonstrate the incredible complexity and diversity of potential resistance mechanisms, and suggest that no single treatment regimen can completely prevent the emergence of drug resistance; indeed, this is applicable not just to CML and TKIs, but perhaps all drug treated cancers. It is therefore critical that as much patient specific information as possible is determined before and during treatment, including patient genomics and transcriptomics, in order to realize precision medicine approaches.

## Materials and methods

### Dasatinib resistant K562 cell line generation

The BCR::ABL1-expressing cell line K562 (American Type Culture Collection (ATCC), Manassas, VA), and the derivative resistance intermediates, were cultured at 37 °C/5% CO_2_ in RPMI-1640 + 10% FCS + L-glutamine (200 mM) + penicillin–streptomycin (5000 U/mL). To obtain dasatinib resistant K562 cells (K562 DasR), K562 cells were exposed to gradually escalating concentrations of dasatinib (0.5–200 nM) as previously described by our laboratory^20^. A diagram displaying the dasatinib escalation method can be found in Fig. 4 and details about time spent in each dasatinib concentration in Supplementary Table 1. Cell viability was monitored by trypan blue exclusion of dead cells. The concentration of dasatinib was increased after > 80% of cells demonstrated survival (dasatinib tolerance) in culture for at least 10 consecutive days. Control cell lines cultured in 0.1% DMSO were maintained in parallel.

### TKI IC_50_ assays and p-CrkL western blotting

To assess TKI susceptibility, the TKI IC_50_ assay was used, determining BCR::ABL1 kinase activity as a measure of CrkL phosphorylation levels^85^. 2 × 10^5^ BCR::ABL1-expressing cells were incubated for 2 h at 37 °C/5% CO_2_ with concentrations of dasatinib ranging 0–5000 nM, in the presence or absence of *ABCG2* inhibitor Ko143 (Sigma-Aldrich, Munich, Germany). Following incubation, cells were lysed in Laemmli’s buffer, before resolution by 12% SDS-PAGE and electrophoretic transfer to PVDF membrane (GE Healthcare, Buckinghamshire, UK) overnight at 65 mA. Western blotting for phosphorylated CT10 regulator of kinase-like (p-CrkL) was performed as previously described^85^. IC_50_ values were determined as the dose of drug required to reduce p-CrkL levels by 50% and are presented as mean + /– SD.

### Transcriptome sequencing and differential expression analysis

RNA was extracted from K562 DasR dose escalation intermediates ranging from 2 to 200 nM DasR, with drug naive K562 and K562 DMSO control lines. Sequencing libraries were prepared using the NEXTFlex Rapid Directional RNA Seq Library Prep Kit (Perkin Elmer, Inc., Waltham, MA), depleting ribosomal and non-coding RNA. Quality control of RNA was performed using the Agilent 2100 Bioanalyzer (Agilent Technologies, Santa Clara, CA), using the Bioanalyzer RNA 6000 Nano assay. Libraries were sequenced on the Illumina MiSeq instrument (Illumina Inc., San Diego, CA), using a 150 cycle to produce 2 × 75 bp paired end reads. Sequencing data was uploaded to the Illumina BaseSpace Sequencing Hub (Illumina Inc.), which was used for FastQC quality control.

Differential expression analysis was performed using the EdgeR pipeline^86^, performed in RStudio version 1.1.447 using R version 3.5.0. Sequencing reads were aligned to the hg19 reference genome using the STAR aligner^87^. Lowly expressed reads (< 10 counts per million) were filtered and samples normalized based on library size. Gene symbol names were annotated from the NCBI database. Hierarchical clustering dendrograms were generated based on the Euclidean distance between pooled sequencing samples, using Ward’s minimum variance method (Supplementary Fig. 1). Based on these results, samples were clustered into discrete groupings to perform differential expression analysis. The K562 10, 15 and 25 nM DasR cell lines (early stage intermediates) were grouped together, for comparison with K562 control samples (drug naïve and DMSO). Similarly, the K562 50 and 200 nM DasR (late stage intermediates) samples were grouped for analysis. Differential expression analysis was then performed using the Linear Models for Microarray and RNA-seq Data (limma) package for R. Differentially expressed genes were visualized by volcano plot, highlighting the top 10 most significantly differentially expressed genes, as well as all genes with logFC > 6.5.

### Geneset enrichment analysis (GSEA)

To test for sets of related genes which may be altered as part of dasatinib resistance, GSEA was performed in early stage and late stage K562 DasR intermediates, comparing with drug naïve controls. Genesets generated by our RNA-seq experiments were compared with the MSigDB Hallmark and C5 gene ontology genesets, using GSEA v4.3.2 software^88,89^. Genesets were stringently filtered by FDR q-value < 0.1.

### Sanger sequencing for BCR::ABL1 kinase domain mutations

The *BCR::ABL1* kinase domain region was amplified using the Expand Long Template PCR System (Roche, Basel, Switzerland) according to manufacturer’s protocols, using Expand Long Template Buffer 3. Sequencing was performed by the Australian Genome Research Facility (AGRF). Sequencing chromatogram files were aligned to *ABL1* sequences using Mutation Surveyor® version 3.30 (SoftGenetics, State College, PA).

### RT-PCR for ABCG2 / BCR::ABL1 gene expression

RNA was obtained from 5 × 10^6^ K562 DasR cell samples in the TRIzol reagent (Invitrogen Life Technologies, Waltham, MA) and extracted using the phenol/chloroform method^90^. Synthesis of cDNA was performed using random hexamers (GeneWorks, Hindmarsh, South Australia) and Superscript II reverse transcriptase (Invitrogen Life Technologies). RT-PCR for *ABCG2* gene expression was performed using the SYBR® Green Real-Time PCR Master Mix system (Qiagen, Dusseldorf, Germany). *ABCG2* expression was normalized to expression of housekeeping gene, *GUSB.* For quantitation of the *BCR::ABL1* transcript levels, RT-PCR was performed using the SYBR® Green Real-Time PCR Master Mix system. For quantitation of the e14a2 (p210 transcript, found in K562 cells, samples were again normalized by expression of housekeeping gene *GUSB*. Gene expression was reported as difference in Ct value (ΔCt) mean + /– SD.

### DNA qPCR for BCR::ABL1 copy number analysis

Genomic quantity of *BCR::ABL1* was determined using the TaqMan FAM-TAMRA system. Primers and probe for amplifying and detecting the *BCR::ABL1* e14a2 intronic breakpoint were used:

- Forward: 5’-TGACCACGGGACACCTTTG-3’
- Reverse: 5’-AGGGTATTTCTGTTTGGGTATGGA-3’
- Probe: 5’-CTGGCCGCTGTGGAGTGGGTTTTATC-3’

These were used in conjunction with TaqMan FAM-TAMRA control primers and probe amplifying genomic *GUSB* sequences:

- Forward: 5’-ATTTTGCCGATTTCATGACTGA-3’
- Reverse: 5’-GACGGGTACGTTATCCCATGAG-3’
- Probe: 5’-ATCCCATGAGCCAAACTGCCACTTACAC-3’

These primer/probe combinations were used with K562 DasR dose escalation intermediate DNA samples, as well as in serial dilutions of *GUSB* and *BCR::ABL1* e14a2 plasmid DNA, allowing relative quantitation of genomic *BCR::ABL1* copy numbers. This was reported as a percentage of *GUSB* quantity.

### Surface expression of ABCG2 transporter

Expression levels of ABCG2 were assessed by flow cytometry as previously described^91^, using PE-conjugated anti-ABCG2 antibody (R&D Systems, Minneapolis, MN). Results were analyzed relative to negative isotype control, PE-conjugated IgG2b (DakoCytomation, Denmark A/S) with an LSRFortessa™ X-20 and FlowJo version 9 (FlowJo LLC, Ashland, OR).

### Intracellular uptake and retention (IUR) assay

To determine the quantity of intracellular dasatinib in leukemic cells, and the dasatinib transport functionality of ABCG2, the intracellular uptake and retention (IUR) assay was performed as previously described^26^. K562 and K562 DasR cells were washed and incubated with 0 or 2 μM C^14^-dasatinib kindly provided by Bristol Myers Squibb (New York, NY), in the presence or absence of 0.5 μM Ko143. This was performed using 2 × 10^5^ cells in 2 mL of RPMI-1640 + 10% FCS, for 2 h at 37 °C. Following incubation, cells were pelleted by centrifugation for 5 min at 6800 rpm then 30 s at 13,000 rpm. Radioactivity of C^14^-dasatinib was determined independently in the supernatant and cell pellet using a Tri-Carb 2810R Liquid Scintillation Counter (Perkin Elmer, Inc., Waltham, MA). The IUR was reported as ng of dasatinib incorporated per 200,000 cells. Results are expressed as paired –/ + Ko143 samples.

### Statistical analysis and figures

Statistical analysis was performed using SPSS (IBM, Armonk, NY), using the unequal variance *t*-test (Student’s *t*-test with Welch’s correction), with spreadsheet manipulation performed using LibreOffice Calc. Figures were produced using the Graphpad Prism 9 (GraphPad Software, Boston, MA).

### Supplementary Information

Supplementary Figure 1.

Supplementary Figure 2.

Supplementary Figure 3.

Supplementary Figure 4.

Supplementary Legends.

Supplementary Table 1.

Supplementary Table 2.

Supplementary Table 2.

## Data Availability

Transcriptome sequencing data is available at the NCBI Gene Expression Omnibus (GEO), under GEO accession number GSE239677.
