# Supplementary figures and images for: The acquisition order of leukemic drug resistance mutations is directed by the selective fitness associated with each resistance mechanism
Source: Sci Rep. 2023 Aug 11;13:13110. doi: 10.1038/s41598-023-40279-2 (PMC10421868; doi:10.1038/s41598-023-40279-2)

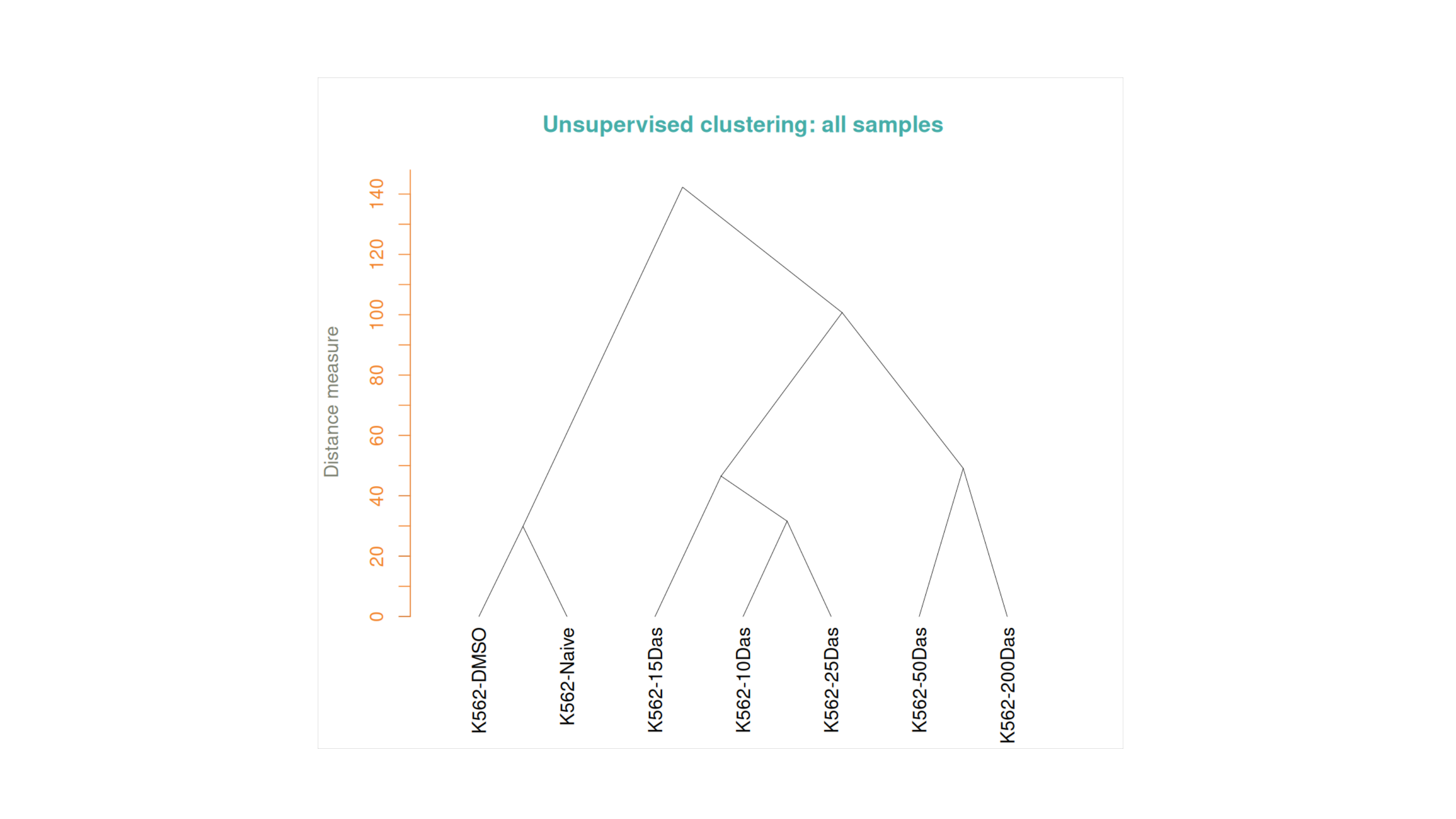

Supplement: Supplementary file 1 — Supplementary Figure 1. [file 41598_2023_40279_MOESM1_ESM.png]

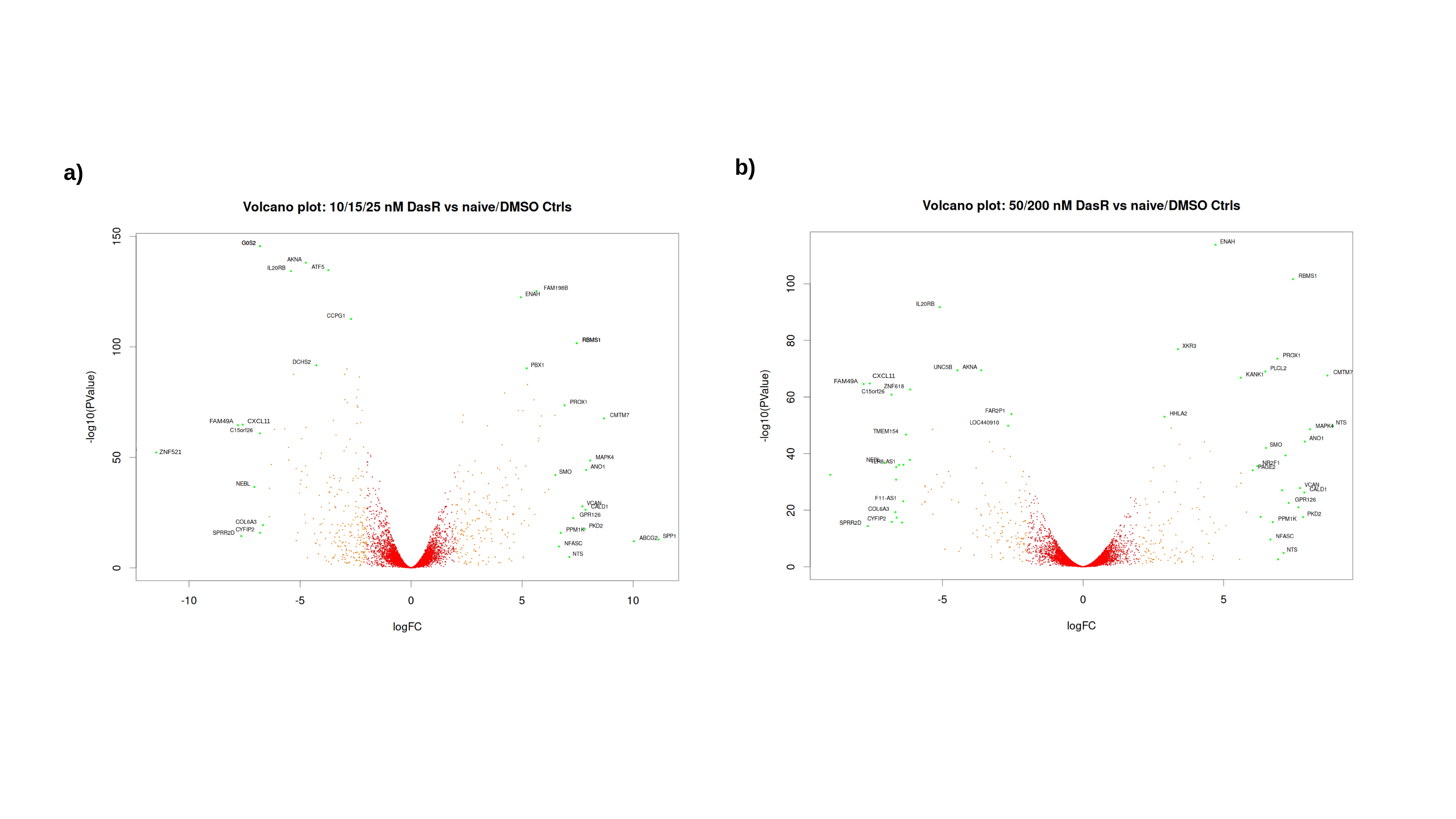

Supplement: Supplementary file 2 — Supplementary Figure 2. [file 41598_2023_40279_MOESM2_ESM.png]

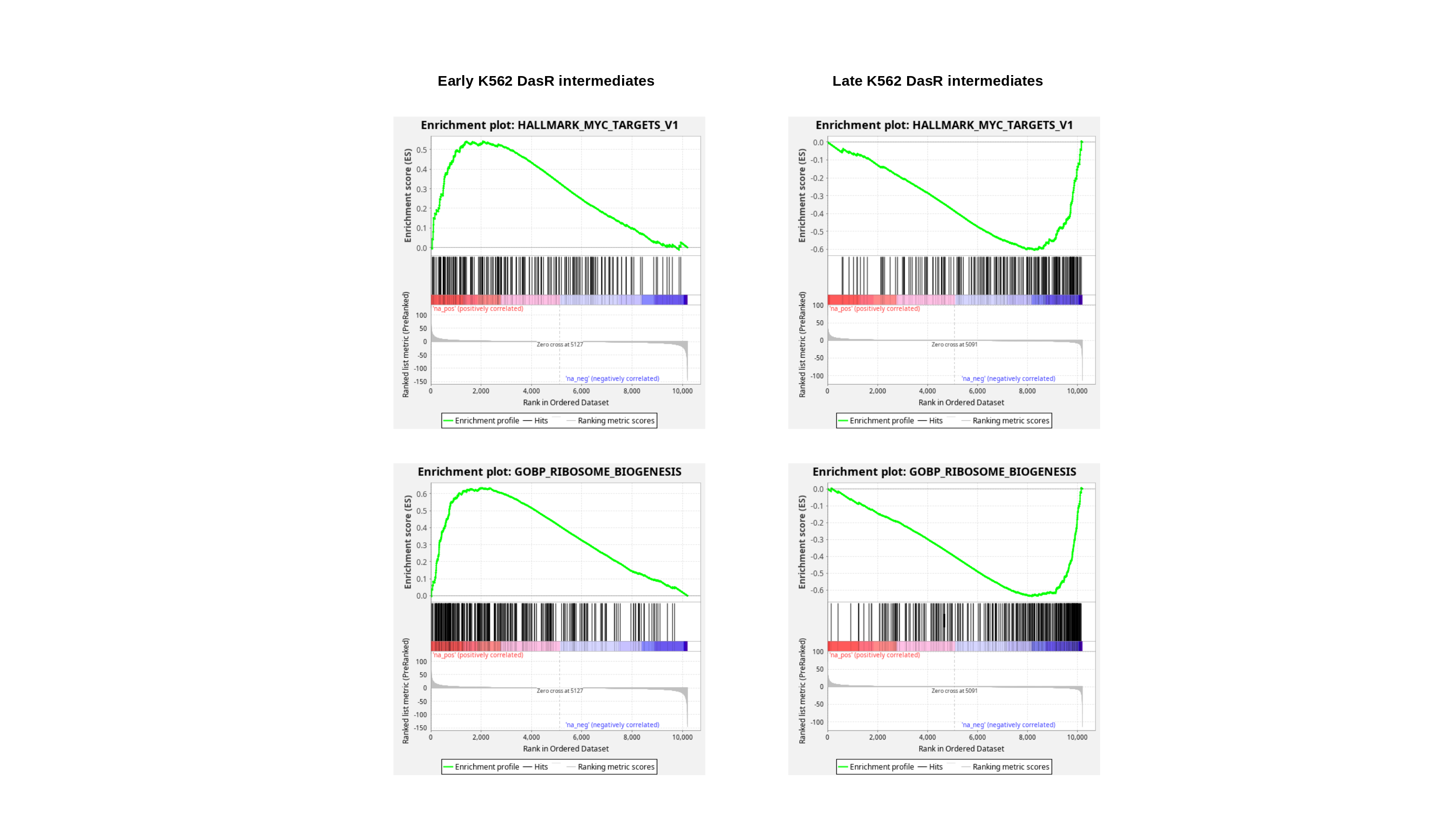

Supplement: Supplementary file 3 — Supplementary Figure 3. [file 41598_2023_40279_MOESM3_ESM.png]

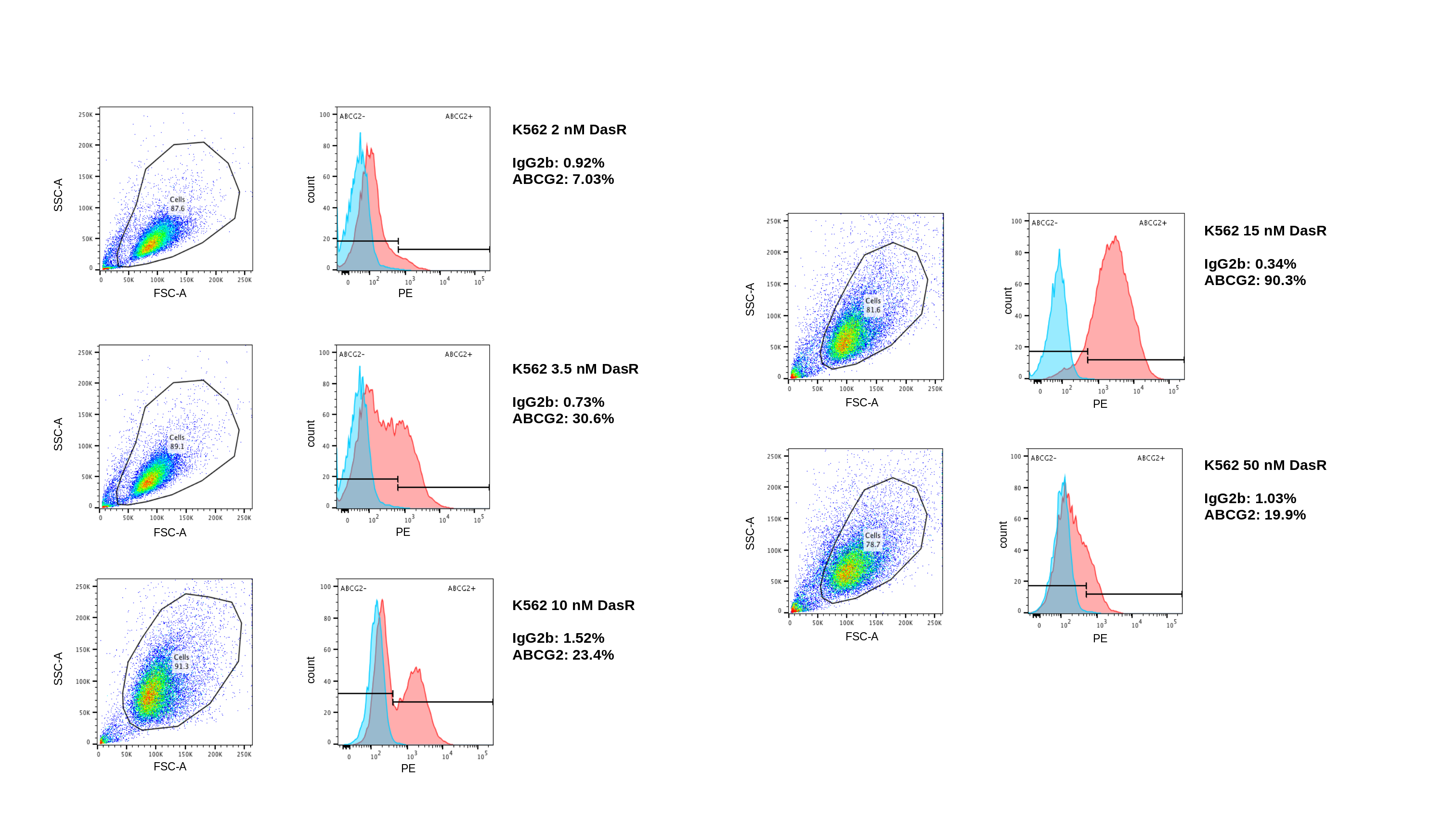

Supplement: Supplementary file 4 — Supplementary Figure 4. [file 41598_2023_40279_MOESM4_ESM.png]
