# Supplementary material for: The acquisition order of leukemic drug resistance mutations is directed by the selective fitness associated with each resistance mechanism
Source: Sci Rep. 2023 Aug 11;13:13110. doi: 10.1038/s41598-023-40279-2 (PMC10421868; doi:10.1038/s41598-023-40279-2)
Supplement: Supplementary file 5 — Supplementary Legends. [file 41598_2023_40279_MOESM5_ESM.docx]

**Supplementary Information**

**Supplementary Figure 1. Unsupervised hierarchical clustering of transcriptome gene expression data grouped samples for differential expression analysis.**

Unsupervised clustering was performed using Ward's minimum variance method, whereby cell lines with similar gene expression profiles cluster together. Results were used to group samples for differential expression analysis; K562 10, 15 and 25 nM DasR (early stage intermediates), 50 and 200 nM DasR (late stage intermediates), K562 DMSO and naive (controls).

**Supplementary Figure 2. Global gene expression identifies several potential mediators of TKI resistance.**

Volcano plots identifying the top 10 most highly differentially regulated genes in a) K562 DasR early and b) late stage intermediates, compared with drug naive controls. Several of the differentially expressed genes in early intermediates played roles in dormancy, stemness and drug resistance.

**Supplementary Figure 3. Geneset enrichment analysis (GSEA) reveals potentially perturbed pathways associated with dasatinib treatment.**

Enrichment plots of the Hallmark MYC targets and C5 ribosome biogenesis genesets, identified by GSEA analysis as concordant with differentially regulated genes in the K562 early and late stage DasR intermediates.

**Supplementary Figure 4. Surface ABCG2 transiently increases over dasatinib dose escalation.**

Representative ABCG2 flow cytometry scatterplots and histograms. Blue histogram indicates positivity for IgG2b isotype control; red histogram for ABCG2. ABCG2 percentage positivity was determined by exclusion of IgG2b isotype positivity.

**Supplementary Table 1. Dasatinib resistance generation timepoints.**

Table summarizing the number of days each intermediate cell line was cultured in a specific dasatinib concentration before escalation to the subsequent dasatinib concentration.

**Supplementary Table 2. Geneset enrichment analysis (GSEA) reveals potentially perturbed pathways associated with dasatinib treatment.**

Tabulated data of the most concordant genesets within the a) Hallmark and b) C5 gene ontology genesets. Genesets were filtered by FDR q-value < 0.1.
